# Integrative species delimitation helps to find the hidden diversity of the leaf-litter frog *Ischnocnema manezinho* (Garcia, 1996) (Anura, Brachycephalidae), endemic to the southern Atlantic Forest

**DOI:** 10.7717/peerj.15393

**Published:** 2023-05-25

**Authors:** Caroline Batistim Oswald, Rafael Félix de Magalhães, Paulo C.A. Garcia, Fabrício R. Santos, Selvino Neckel-Oliveira

**Affiliations:** 1 Programa de Pós-Graduação em Zoologia, Universidade Federal de Minas Gerais, Belo Horizonte, Brazil; 2 Departamento de Ciências Naturais, Universidade Federal de São João del-Rei, São João del-Rei, Brazil; 3 Departamento de Ecologia e Zoologia, Universidade Federal de Santa Catarina, Florianópolis, Brazil; 4 Departamento de Genética, Ecologia e Evolução, Universidade Federal de Minas Gerais, Belo Horizonte, Brazil

**Keywords:** Conservation genetics, Bioacoustics, Morphometry, Phylogeography, Species delimitation, Red Lists, Endangered

## Abstract

**Background:**

The delimitation of cryptic species is a challenge for biodiversity conservation. Anurans show high cryptic diversity levels, and molecular species delimitation methods could help identify putative new species. Additionally, species delimitation approaches can provide important results for cryptic species conservation, with integrative methods adding robustness to results. *Ischnocnema manezinho* was described from Santa Catarina Island (SCI), southern Brazil. More recently, some inventories indicated continental populations supposedly similar in morphology to it. If these records are confirmed as *I. manezinho,* it would likely change its endangered status on National Red List, removing the species from conservation agendas. We investigated the threatened frog *Ischnocnema manezinho*, to evaluate if the continental populations belong to this species or if they form an undescribed species complex.

**Methods:**

We used coalescent, distance, and allele-sharing-based species delimitation methods and integrative analyses of morphometric and bioacoustics traits to test evolutionary independence between *I. manezinho* from SCI, Arvoredo Island, and continental populations.

**Results:**

*Ischnocnema manezinho* is restricted to Santa Catarina Island, while the five remaining lineages should be further investigated through a taxonomic review. Our results point to a small geographic range of *Ischnocnema manezinho*. Additionally, the species occurs in isolated fragments of forest in SCI surrounded by expanding urban areas, confirming its status as Endangered. Thus, the protection and monitoring of *I. manezinho* and the taxonomic description of the continental and Arvoredo Island candidate species should be priorities.

## Introduction

Integration between conservation and taxonomy is essential for the accurate categorization of species in Red Lists (Mace, 2004; Magalhães et al., 2018). Splitter species delimitations, which describe population varieties as full species, could list populations of least concern taxa in threatened categories. The independent management of them could result in inbreeding depression and/or misallocation of resources for their protection (Frankham et al., 2012; Stanton et al., 2019). Conversely, lumper delimitations, that merge two or more independent lineages under a single name, could downlist species from extinction risk categories, and managing them together under the same name can lead to outbreeding depression and/or extinction of hidden diversity (Frankham et al., 2012; Delić et al., 2017). So, splitter and lumper species delimitation can influence conservation practice, with the potential to refute official priorities on Red Lists (Angulo & Icochea, 2010; Fišer, Robinson & Malard, 2018; Struck et al., 2018). This is worrying for species-focused conservation because until evolutionarily independent lineages (*i.e.,* species *sensu* Simpson, 1951) are known as formal taxa, they do not receive funds for their conservation, even under threat of extinction (Bickford et al., 2007; Angulo & Icochea, 2010). Thus, the delimitation of species in operational taxonomic units and the understanding of their geographic distribution is an essential question for taxa-targeted conservation, especially for threatened species (Funk, Caminer & Ron, 2012; Delić et al., 2017; Magalhães et al., 2018).

Although morphological comparisons have been used as a primary source of information on taxonomic studies, DNA data can also provide important evidence for systematics and conservation, increasing precision and replicability in taxonomic decisions (Fujita & Leaché, 2011; Fujita et al., 2012). DNA-based species delimitation provides a standardized way of testing distinct taxonomic scenarios (Flot, Couloux & Tillier, 2010; Fontaneto, Flot & Tang, 2015). Among the most widely used models in the literature, the multi-species coalescent (MSC) uses generalizations of the Wright-Fisher model of genetic drift to test the genealogical boundaries between distinct lineages (Knowles & Carstens, 2007). MSC methods consider the uncertainties and inconsistencies between gene trees resulting from random processes occurring at the population level, as different coalescent genealogies of independent loci (Knowles & Kubatko, 2011). However, these methods often overestimate the number of delimited species (Sukumaran & Knowles, 2017; Leaché et al., 2019). Other methods, like distance- and allele-sharing-based approaches can underestimate the number of species in several situations, such as in cases of high speciation and low mutation rates (Dellicour & Flot, 2018). Therefore, a recommended practice is the use of independent methods to check consistency in species delimitation (Carstens et al., 2013; Fišer, Robinson & Malard, 2018) plus integrative data, such as ecological and behavioral traits (Padial et al., 2010; Fujita et al., 2012).

Some taxonomic groups seem to show high diversification and genetic structure than others (Trontelj & Fišer, 2009; Pérez-Ponce De León & Poulin, 2016). This seems to be the case of brachycephalid frogs, which contain two genera, *Brachycephalus* Fitzinger, 1826 and *Ischnocnema* Reinhardt & Lütken, 1862 (Heinicke, Duellman & Hedges, 2007). Many phylogeographical studies have discovered deep population structures in some species of this family, suggesting numerous new species with restricted geographic distribution from former widespread ones (Gehara et al., 2013; Gehara et al., 2017; Thomé et al., 2020). This is especially evident in the genus *Ischnocnema*, in which several morphologically similar species or candidate species are differentiated by call traits (Kwet & Solé, 2005; Gehara et al., 2013; Taucce, Canedo & Haddad, 2018).

*Ischcnocnema* is distributed along the Atlantic Forest, from northeastern to southern Brazil, northeastern Argentina, and possibly Paraguay (Hedges, Duellman & Heinicke, 2008; Frost, 2023). The genus comprises 39 species (Frost, 2023). Around 15% of them have been described in the last five years (*e.g.*, Taucce et al., 2019; Silva-Soares et al., 2021), indicating a taxonomic shortfall in the genus. *Ischnocnema manezinho* (Garcia, 1996) is a leaf-litter frog described from Santa Catarina Island (Garcia, 1996). Additionally, some studies and inventories have recorded continental populations attributed to *I. manezinho* due to similarities in external morphology (Wachlevski-Machado, 2011; Canedo & Haddad, 2012; Taucce, Canedo & Haddad, 2018; Taucce et al., 2018), but no comparative study has been carried out between the two regions. The species is categorized as Endangered in the Brazilian Red List (MMA, 2022), due to its restricted distribution, loss of area, quality, and fragmentation of habitat (ICMBio, 2023). For the assessment, Brazilian Red List authors considered only the populations of the Island of Santa Catarina (ICMBio, 2023). However, if the continental populations are confirmed as conspecific, the species would be of lesser concern regarding its extinction risk. Thus, we evaluated the hypothesis of the species *I. manezinho* is restricted to Santa Catarina Island, and the continental populations belong to new candidate species versus *I. manezinho* having a wide range, occupying both regions. Specifically, we aim to evaluate the geographic limits of *Ischnocnema manezinho*, suggesting conservation and taxonomic research priorities.

## Materials and Methods

### Sampling

We obtained 41 tissue samples and 83 specimens identified as *I. manezinho* in distinct zoological collections and supplemented them with 20 tissue samples and 24 specimens of *I. manezinho sensu lato* (*i.e.,* including all individuals morphologically identified as *I. manezinho*) collected in field expeditions. We conducted field expeditions in the spring and summer between October 2014 and December 2016 in the eastern region of the State of Santa Catarina, including locals of possible distribution and locals with any reports of the presence of *I. manezinho sensu lato* (Table S1). We also collected one specimen of *I. sambaqui* found co-occurring with *I. manezinho sensu lato* in São Francisco do Sul, Santa Catarina, Brazil (UFMG 19194; Table S1). We searched actively *Ischnocnema manezinho sensu lato* in areas with rocky outcrops in the southern Atlantic Forest from dusk to night. We collected the individuals under governmental collection permits numbers 47781 and 45770 provided by the Instituto Chico Mendes de Conservação da Biodiversidade (ICMBio), and 12/2015 provided by Instituto do Meio Ambiente de Santa Catarina (IMA). We euthanized anurans with 5% xylocaine, and we preserved tissue samples in 96% ethanol and specimens in 70% ethanol, after fixing them in 3.7% formalin. We deposited all biological samples in the Herpetological Collections of Universidade Federal de Santa Catarina (CHUFSC) and Centro de Coleções Taxonômicas - Universidade Federal de Minas Gerais (CCT-UFMG).

#### Genetic data

We included tissues from 61 individuals of *I. manezinho sensu lato* (Table S1) for which we sequenced three nuclear (nDNA) [Fibrinogen A alpha-polypeptide, intron 1 (*α*-fib); Beta-fibrinogen, intron 7 (*β*-fib); Chemokine Receptor 4 (*cxc*)] and one mitochondrial (mtDNA) [Cytochrome B (cyt-*b*)] gene fragments. We also sequenced DNA fragments from three specimens of *I. sambaqui* (Castanho & Haddad, 2000) and two of *I. henselii* (Peters, 1870) (Table S1) as outgroups for phylogenetic analyses since *I. sambaqui* is recovered as sibling species to *I. manezinho sensu lato* (Canedo & Haddad, 2012; Taucce et al., 2018). The inclusion of them in species tree analysis allowed us to verify the monophyly of *I. manezinho sensu lato*.

We carried out the genomic extraction following a standard phenol-chloroform protocol (Sambrook & Russel, 2001) and obtained the gene fragments *via* polymerase chain reaction (PCR), using specific primers (Table S2). We performed the PCR in a 15 µL reaction volume containing: 30 ng of genomic DNA, 1 × Buffer, 1.25 µM each primer, 2.5 mM MgCl_2,_ 0.72 µg bovine serum albumin (BSA), 3mM dNTPs, and 0.625 U Platinum™*Taq* DNA polymerase (Thermo Fisher Scientific). We performed the amplifications as one initial denaturation at 94 °C for 5 min, followed by 40 cycles (35 cycles for cyt-*b*) [denaturation at 94 °C for 30 s, variable melting temperatures and times between fragments (56−62 °C by 40–60 s; Table S2), extension at 72 °C for 1 min/1,000 bp], and a final extension stage at 72 °C for 7 min. We purified the DNA amplicons using polyethylene glycol 20% protocol (Santos, Santos & Silveira, 2015) and sequenced them in both strands, using the same amplification primers from PCR (Table S2) and BigDye Terminator v. 3.1 kit (Life Technologies™), in a Sanger automatized sequencer ABI 3130XL (Applied Biosystems™).

We used SeqScape v. 2.6 software (Applied Biosystems, Waltham, MA, USA) to assemble, check, and edit the sequence fluorograms. We aligned the edited sequences using the ClustalW algorithm (Larkin et al., 2007), implemented in MEGA11 software v. 11.0.9 (Tamura, Stecher & Kumar, 2021). We phased the heterozygous nuclear sequences with PHASE v. 2.1.1 (Stephens, Smith & Donnelly, 2001; Flot, 2010), using a 70% posterior probability (PP) threshold to consider haplotype phases solved. Some individuals showed heterozygous indels for *α*-fib and *β*-fib. In these cases, we phased sequences of individuals with heterozygous indels in the Indelligent v. 1.2 web tool (Dmitriev & Rakitov, 2008a; Dmitriev & Rakitov, 2008b).

#### Morphometric data

**Figure 1:**
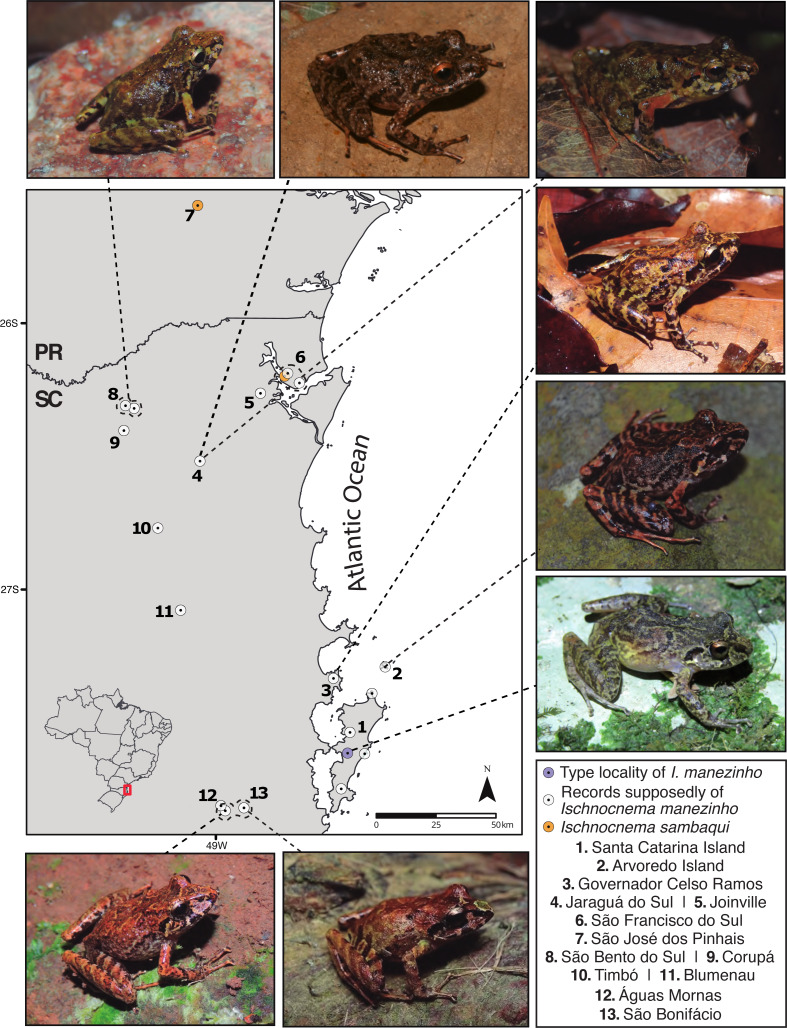
Sampling map with some representatives of *Ischnocnema manezinho sensu lato* in each location, considering only genetic data. PR = Paraná State and SC = Santa Catarina State; Numbers represent municipalities in both states. Photo credit (clockwise): Caroline B. Oswald (CBO); Thais Condez; CBO; Ivo Ghizoni Jr.; CBO; Vitor Carvalho-Rocha; Leandro Drummond; CBO.

We examined morphometric data from 107 males of *I. manezinho sensu lato* from eastern Santa Catarina state, southern Brazil (Fig. 1; Table S1). For municipalities where we sampled more than one genetic lineage (see Results), we only measured specimens that were also genetically evaluated. For each individual, we took 20 body measurements (Table S3), including snout-vent length (SVL), head length (HL), head width (HW), eye diameter (ED), eye-nostril distance (EN), snout-nostril length (NS), internarial distance (IND), upper eyelid width (UEW), distance between the anterior margins of eyes (AMD), interorbital distance (IOD), tympanum diameter (TD), forearm length (FLL), forearm breadth (FAW), hand length (HAL), thigh length (THL), tibia length (TL), tarsus length (TSL), foot length (FL), finger IV disk width (Fin4DW), and toe IV disk width (Toe4DW). We followed Watters et al. (2016) for morphometric terminology and definitions, except for AMD, in which we followed Garcia, Vinciprova & Haddad (2003). We performed the measurements in preserved specimens with a digital caliper with 0.1 mm precision, on the right side in the dorsal view. In cases of impossibility due to poor preservation or malformation, we measured the left side.

We used only morphometric traits of males in the comparative analysis since most anuran species exhibit sexual dimorphism in body size (Nali et al., 2014). We identified males by the presence of vocal slits under the tongue. We used ratios of measurements over SVL, to correct the size effect, following Goutte et al. (2022). Before morphometric analysis, we eliminated the correlated variables. For this, we calculated the variance inflation factor (VIF) for all variables with the ‘usdm’ v. 1.1-18 R package through the *vifstep* function and threshold of five (Naimi et al., 2014). As a result, we eliminated the variables TL and Toe4DM from the final morphometric matrix.

#### Bioacoustics data

We analyzed 323 calls of 36 males for *Ischnocnema manezinho sensu lato* with the Raven Pro 1.5 Beta software (Bioacoustics Research Programm, 2013), using the following configurations: window type = Hamming, window size = 256 samples, bandwidth 3 dB filter = 224 Hz, overlap = 89.8%, DFT size = 256 samples, grid spacing = 172 Hz, brightness = 50% and contrast = 50%. Temporal and spectral parameters were measured on oscillogram and spectrogram, respectively. We measured the call duration (CD), dominant frequency (DF), low frequency (LF), high frequency (HF), number of notes (NN), and note rate (NR) for each call. We adopted the note-centered approach and followed Köhler et al. (2017) for call, note, note rate, call duration, and dominant frequency definitions. We constructed the final dataset for statistical analysis with the mean values of each individual (Table S4). Before bioacoustics comparisons, we eliminated the correlated variables. For this, we calculated the variance inflation factor (VIF) for all variables with the ‘usdm’ v. 1.1-18 R package through the *vifstep* function and threshold of five (Naimi et al., 2014). As a result, we eliminated the variables CD and HF from the final bioacoustics matrix.

### Species delimitation

We conduct a *ϕ*-test for recombination in SplitsTree v. 4.14.8 (Bruen, Philippe & Bryant, 2006; Huson & Bryant, 2006) and Tajima’s D neutrality test in the ‘pegas’ v. 0.13 R package (Tajima, 1989; Paradis, 2010) to test lack of recombination and neutrality in nDNA fragments for coalescent analysis. The dataset summary statistics were estimated in the ‘ape’ v. 5.0 and the ‘pegas’ R packages (Paradis, 2010; Paradis & Schliep, 2019; R Core Team, 2022).

We did the delimitation analyses in two steps: the discovery methods and then the validation models (Ence & Carstens, 2011; Carstens et al., 2013). For the discovery step, we adopted one distance-based and one gene tree-based method to mtDNA data, and one haplotype sharing-based method to nDNA data.

First, we applied the Assemble Species by Automatic Partitioning (ASAP) method and the multi-rate Poisson Tree Processes (mPTP) method. We used the online ASAP version (Puillandre, Brouillet & Achaz, 2021; https://bioinfo.mnhn.fr/abi/public/asap/), with Kimura (K80) distance model. We set the average ratio between transitions (ts) and transversions (tv) at 0.18 and other parameters were left as default. We estimated this value (tv/ts) using the Kimura 2-parameters (K80) model (Kimura, 1980) and removing missing nucleotides for each sequence pair (pairwise deletion option) in MEGA X v. 10.2.6 Kumar et al., 2018). We performed the ASAP analysis with all mitochondrial sequences available. We select the best asap-score to define the optimal number of species partitions. To perform mPTP species delimitation analysis, we inferred unique cyt-*b* haplotypes with ‘haplotypes’ v. 1.1.2 R package (Aktas, 2020; R Core Team, 2022) using the method ‘sic’. Then, we inferred a maximum likelihood tree using RAxML v. 8.2.10 from this haplotype-reduced data set, spending the GTRGAMMA substitution model and 1000 replicates to estimate the bootstrap support values (Stamatakis, 2014). We ran the mPTP analysis on the online platform (Kapli et al., 2017; https://mptp.h-its.org/#/tree).

We ran the nuclear discovery method through the haplowebs approach (Flot, Couloux & Tillier, 2010). For this, we implemented the method with a conspecificity matrix (CoMa) in Haplowebmaker online tool (Flot, Couloux & Tillier, 2010; Spöri & Flot, 2020). We made this analysis to search for groups of individuals that form reciprocally exclusive allelic pools, which can be considered reproductively isolated (Flot, Couloux & Tillier, 2010). We constructed the haplowebs through the median-joining algorithm, using singletons and considering indels as a 5th character state. We assume that indels larger than 1-bp were the result of a single mutational event and, in these cases, we represented them as 1-bp indels. Furthermore, to visualize the nDNA haplotypes relationship, we calculated an nDNA multilocus distance matrix using the genpofad algorithm implemented in POFAD v. 1.07 software, with the additive method to infer missing nucleotides and sequences (Joly & Bruneau, 2006; Joly, Bryant & Lockhart, 2015). We used the NeighborNet algorithm ( Bryant & Moulton, 2004) in SplitsTree v. 4.14.8 software (Huson & Bryant, 2006) to convert the resultant pairwise distances matrix into a network.

We compared the lineages delimited in each discovery method and considered as input for the validation step only the independent units recovered in all of them. We implemented the validation through the Bayesian Phylogenetics and Phylogeography method (BPP) v. 4, using species delimitation and tree estimation concomitantly (analysis A11) (Yang & Rannala, 2014). For this analysis, we removed the *β*-fib fragment due to the evidence of recombination (see Results). We combined two distinct gamma prior [theta (Θ) and tau (*τ*) parameters] and two initial trees for the analyses. First, we ran BPP with the prior values estimated by Minimalist BPP (https://brannala.github.io/bpps/) based on our dataset (Θ = 0.019 and *τ* = 0.48) and one run with a diffuse prior value (Θ = 0.19 and *τ* = 4.8). The other finetune parameters were assigned for automatic adjustment by Minimalist BPP for both runs. We conducted all analyses with 2.5 × 10^5^ generations and 2 × 10^3^ burn-in.

Based on our species delimitation and the current taxonomy, we named the lineages as unconfirmed candidate species (UCS), plus a sequential number (Padial et al., 2010) in the following analysis. Individuals from the type-locality, and individuals that clustered with them in our analysis, were identified as *I. manezinho.*

Lastly, we estimated uncorrected mtDNA pairwise p-distances for comparative purposes on MEGA11 v. 11.0.9 (Tamura, Stecher & Kumar, 2021), using pairwise deletion for comparisons between fragments with missing nucleotides. We calculated the p-distances only for *I. manezinho sensu lato.*

### Morphometric and Bioacoustics congruence analyses

We subjected the morphometric and bioacoustics data, separately, to a non-parametric Multivariate Analysis of Variance (NP- MANOVA) in the ‘RRPP’ v. 1.3.1 R package (Collyer & Adams, 2018; Collyer & Adams, 2019; R Core Team, 2022) to verify differences between putative species. We implemented a post hoc pairwise test in the same R package, to check which groups differ from each other.

### Species-tree and divergence time estimation

We generated a dated species tree in starbeast3 v. 1.1.4, with a birth-death tree prior implemented in BEAST2 v. 2.7.2 (Gernhard, 2008; Bouckaert et al., 2019; Zhang & Drummond, 2020; Douglas, Zhang & Bouckaert, 2021; Douglas, Jimenez-Silva & Bouckaert, 2022) to estimate the most recent common ancestor of the candidate species and divergence times of cladogenetic events. Due the absence of fossil records, we calibrated the tree using four calibration points from Hime et al. (2021) for the Brachycephaloidea superfamily. These authors used 19 fossil-based calibrations also used in other divergence time estimates (Feng et al., 2017). So, for this analysis, we included cyt-*b* and *cxc* sequences available in GenBank (Sayers et al., 2020) for the families Ceuthomantidae (*Ceuthomantis smaragdinus*), Eleutherodactylidae (*Eleutherodactylus coqui*), non-*Ischnocnema* Brachycephalide (*Brachycephalus epphipium*), Craugastoridae (*Craugastor podiciferus*), and Strabomantidae (*Pristimantis thymelensis* and *Phrynopus bracki*) as outgroups. We used normally distributed priors for the divergence within Strabomantidae [mean = 40.44 Ma, sigma = 2.17], defining a 95% range of 36.2–44.7 Ma; between Craugastoridae and Strabomantidae [mean = 43.56 Ma, sigma = 2.17], and 95% range of 39.3–47.8 Ma; between Eleutherodactylidae and Craugastoridae+Strabomantidae+Brachycephalidae [mean = 45.61 Ma, sigma = 2.2], and 95% range of 41.3–49.9 Ma; between Ceuthomantidae and Eleutherodactylidae+Craugastoridae+Strabomantidae+Brachycephalidae [46.98 Ma, sigma = 2.2], and 95% range of 42.7–51.3 Ma; and for the crown age of Brachycephaloidea [mean 54.21, sigma = 2.15], defining 95% range of 50.0–58.4 Ma. We used the ‘bModelTest’ package v. 1.3.2 (Bouckaert & Drummond, 2017) to co-estimate the nucleotide substitution model for all fragments in the species tree estimation.

We analyzed two replicates, with 1 × 10^8^ generations, 1.8 × 10^4^ thinning, and 5% burn-in each run. We combined the results of all runs using LogCombiner v. 2.7.2 (Bouckaert et al., 2019) and annotated the MCC tree with TreeAnnotator v. 2.7.2 (Bouckaert et al., 2019). We checked the stationarity and convergence of BEAST analyses using Tracer v.1.7.2 (Rambaut et al., 2018) through a visual inspection of adequate mixing and effective sample sizes (ESS >200) of the estimated parameters.

## Results

### Species delimitation

**Figure 2:**
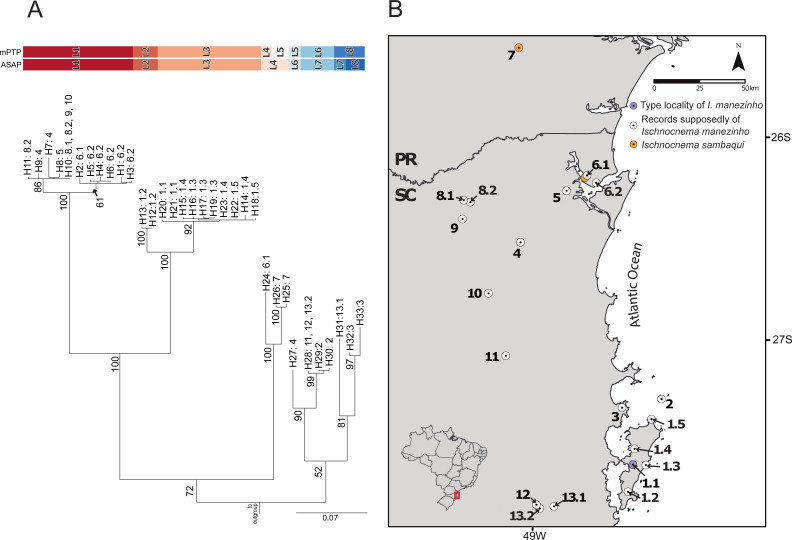
Mitochondrial species delimitation with the respective haplotype distribution. (A) Mitochondrial gene tree estimated by RAxML and species delimitation generated by ASAP and mPTP methods (colorful columns). The outgroup was removed of the visualization. The numbers above the nodes indicate bootstrap values, colorful columns represent distinct lineages by each method, and the names in the terminal branches correspond to the unique haplotypes (Table S1) with their geographic distribution corresponding in B. (B) Geographic distribution of the unique haplotypes of *Ischnocnema manezinho sensu lato*. PR = Paraná State and SC = Santa Catarina State. The map numbers represent the sampling locality (as mentioned in Fig. 1, with some details): 1. Santa Catarina Island [1.1. Cachoeira do Poção, 1.2. Lagoa do Peri, 1.3. Ponta do Gravatá, 1.4. Unidade de Conservação Ambiental Desterro, 1.5. Praia Brava]; 2. Arvoredo Island; 3. Governador Celso Ramos; 4. Jaraguá do Sul; 5. Joinville; 6. São Francisco do Sul [6.1. Morro do Cantagalo, 6.2. CEPA Vila da Glória]; 7. São José dos Pinhais; 8. São Bento do Sul [8.1. Estrada Saraiva, 8.2. CEPA Rugendas]; 9. Corupá; 10. Timbó; 11. Blumenau; 12. Águas Mornas; 13. São Bonifácio [13.1 Morro das Pedras; 13.2 Gruta São José].

The best ASAP partition had an ASAP-score equal to 2.50 and identified eight lineages with a distance threshold of 9.32% (Table S6; Fig. S1). Cyt-*b* presented 33 distinct haplotypes, almost all of them are endemic to the localities where they were sampled except for haplotypes H10 and H28, found in three non-neighboring localities each (Fig. 2; Table S1). The mPTP analysis also identified eight lineages, with a different composition from ASAP. In contrast to the ASAP results, lineage 4 was split into two distinct lineages (mPTP –Lineages 4 and 5) and the diverged lineages 7 and 8 from ASAP were grouped into one lineage (Lineage 8; Fig. 2, Fig. S1). Two distinct mitochondrial lineages were recovered from Santa Catarina Island in both approaches, one from the center/north (Lineage 2) and another from the south (Lineage 3) of the island (Fig. 2, Fig. S1).

**Figure 3:**
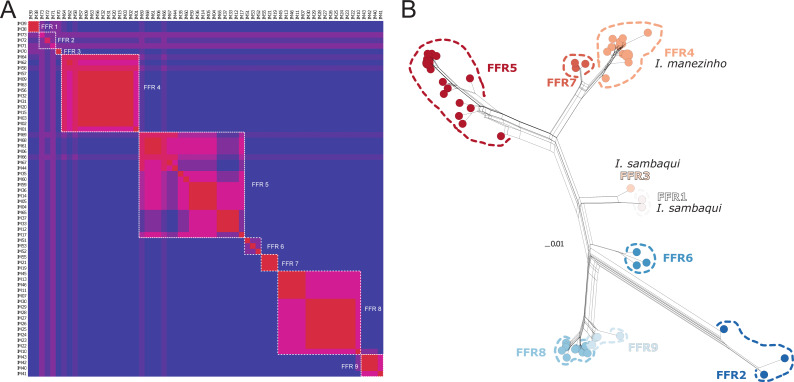
Nuclear species delimitation and relathionship of the haplotypes. (A) Heat map of conspecificity matrix (CoMa) of haplowebs. The Color scale indicates the conspecificity scores, varying from red to purple representing highest to lowest scores, respectively. Pink tones represent intermediate scores. Dashed white quadrates delimit fields for recombination (FFR) identified by the method. IM codes correspond to individuals sampled, details in Table S1. (B) Multi-loci nuclear neighbor-net. The scale bar represents standardized genetic distances, and colors represent the distinct lineages delimited by Haplowebs.

There were 12 single fields for recombination (sl-FFR; *sensu* Flot, Couloux & Tillier, 2010) in *α-*fib, 18 in *β-*fib, and 12 in *cxc* (Fig. S2; Table S7). The delimitation scenario of haplowebs and CoMa was subtly more splitter than mitochondrial approaches, identifying nine lineages. Haplowebs recovered five lineages also present in mtDNA delimitations and indicated the splitting of the other two lineages. In the Haplowebs, individuals of Governador Celso Ramos (FFR2) and São Bonifácio-Morro das Pedras (FFR6; Fig. 2) correspond to two independent lineages as in ASAP and the individuals of *Ischnocnema sambaqui* (FFR1 and FFR3) also correspond to two independent lineages as in mPTP (Figs. 2, 3A). The nDNA loci show exclusive and well-differentiated clades agreeing with the mtDNA lineages (Fig. 3B). The lack of individuals in the network’s inner branches suggests the absence of gene flow among lineages (Fig. 3B).

We did not find deviation from neutrality in any of the markers since they all showed non-significant values of Tajima’s D (Table S5). On the other hand, we find in the *ϕ*-tests statistically significant evidence for recombination in *β*-fib (Table S5). So, we removed the *β-*fib fragment from coalescent analyses.

The validation step (BPP) confirms the seven lineages delimited in the consensus of discovery methods for all replicates, with high statistic support (PP = 1), regardless of *θ* and *τ* combinations and initial tree (Table 1). The two replicates of distinct combinations of gamma prior [Θ and *τ* parameters] and initial tree resulted in the same best tree (*i.e.,* ((*I. sambaqui*, (L6, (L4, L5))), (L1, (*I. manezinho*, L2)))) with high statistic support (Table 1). *Ischnocnema manezinho sensu lato* (*i.e.,* including all individuals morphologically identified as *I. manezinho*) is paraphyletic since *I. sambaqui* is nested within the complex. BPP validated the presence of one independent lineage in Santa Catarina Island besides *Ischnocnema manezinho* of type-locality.

**Table 1:** Posterior probabilities for each delimited candidate species and tree estimated for each gamma prior combination for theta (Θ) and tau (*τ*) in BPP. The first and second lines refer to each two-independent replicate with distinct input tree.

Priors	Species probability							Best tree	Tree probability
	***I. manezinho***	**UCS1**	**UCS2**	**UCS3**	**UCS4**	**UCS5**	***I. sambaqui***		
Θ = 0.019; *τ* = 0.48	1	1	1	1	1	1	1	((*I. sambaqui*, (L6, (L4, L5))), (L3, (*I. manezinho*, L2)))	0.98750
	1	1	1	1	1	1	1	((*I. sambaqui*, (L6, (L4, L5))), (L3, (*I. manezinho*, L2)))	0.98736
Θ = 0.19; *τ* = 4.8	1	1	1	1	1	1	1	((*I. sambaqui*, (L6, (L4, L5))), (L3, (*I. manezinho*, L2)))	0.98866
	1	1	1	1	1	1	1	((*I. sambaqui*, (L6, (L4, L5))), (L3, (*I. manezinho*, L2)))	0.98930

Inter-lineage p-distances confirm a high mtDNA divergence between the unconfirmed candidate species, varying from 5.58% (between UCS1 and *Ischnocnema manezinho*) to 28.77% (between UCS2 and UCS5). UCS5 showed 7.7% of intra-lineage distance, which overlapped with some inter-lineage distances (Table 2).

**Table 2:** Average uncorrected p-distances for the cyt-*b* gene within (bold) and between unconfirmed candidate species (UCS) and *Ischnocnema manezinho*.

	***Ischnocnema manezinho***	**UCS1**	**UCS2**	**UCS3**	**UCS4**	**UCS5**
***Ischnocnema manezinho***	**0.0038**					
**UCS1**	0.0558	**0.0010**				
**UCS2**	0.2243	0.2143	**0.0173**			
**UCS3**	0.2578	0.2606	0.2752	**0.00**		
**UCS4**	0.2714	0.2483	0.2707	0.0710	**0.0035**	
**UCS5**	0.2630	0.2587	0.2877	0.1620	0.1646	**0.0773**

### Morphometric and bioacoustics congruence analyses

Despite the significant result of morphometric MANOVA (*F* = 31.65, *Df* = 5, *p* < 0.05; Table 3), *Ischnocnema manezinho* is only morphometrically distinct from UCS2 and from UCS4. All other genetic lineages were unable to be morphometrically differentiated from the type-locality. Additionally, the unconfirmed candidate species pairs UCS2 and UCS4, UCS3 and UCS4, and UCS4 and UCS5 are morphometrically distinct from each other (Table 3).

**Table 3:** Non-parametric MANOVA results showing differences in morphometric data of unconfirmed candidate species and *Ischnocnema manezinho*.

	**Df**	**Residual Df**	**SS**	**Residual SS**	**Rsq**	**F**	**Z**	**Pr (>F)**
**Lineages**	5	101	1092.10	696.93	0.61	31.65	8.71	1 × 10^−4^

**Pairwise: distance between means - d (Z)**						
	***I. manezinho***	**UCS1**	**UCS2**	**UCS3**	**UCS4**	**UCS5**
***I. manezinho***	–					
**UCS1**	0.09 (-2.00)	–				
**UCS2**	4.31 (3.14)^*^	4.40 (1.40)	–			
**UCS3**	0.25 (-1.46)	0.34 (-1.49)	4.08 (1.50)	–		
**UCS4**	4.34 (3.29)^*^	4.26 (1.36)	8.65 (4.87)^*^	4.57 (1.70)^*^	–	
**UCS5**	3.29 (1.19)	3.38 (0.63)	1.02 (-0.40)	3.07 (0.61)	7.64 (2.86)^*^	–

**Notes.**

* *p* < 0.05.

In the same way, bioacoustics differences had significant results (*F* = 35.60, *Df* = 4, *p* < 0.05; Table 4). However, only UCS4 could be differentiated from *Ischnocnema manezinho* by the parameters analyzed here (Table 4). Additionally, the unconfirmed candidate species pairs UCS2 and UCS4 also showed acoustic differences between them (Table 4). UCS1 could not be compared due to the lack of available call records.

**Table 4:** Non-parametric MANOVA results showing differences in bioacoustics data of the unconfirmed candidate species and *Ischnocnema manezinho*.

	**Df**	**Residual Df**	**SS**	**Residual SS**	**Rsq**	**F**	**Z**	**Pr(>F)**
**Lineages**	4	31	14846052	3231562	0.82	35.60	8.11	1 × 10^−4^

**Pairwise: distance between means - d (Z)**					
	***I. manezinho***	**UCS2**	**UCS3**	**UCS4**	**UCS5**
***I. manezinho***	–				
**UCS2**	398.59 (0.81)	–			
**UCS3**	654.22 (0.05)	1,042.78 (1.08)	–		
**UCS4**	1,123.42 (3.09)^*^	1,521.56 (3.98)^*^	509.08 (-0.42)	–	
**UCS5**	540.26 (0.41)	906.21 (1.32)	187.15 (-1.53)	691.17 (0.86)	–

**Notes.**

* *p* < 0.05.

### Species-tree and divergence time estimations

**Figure 4:**
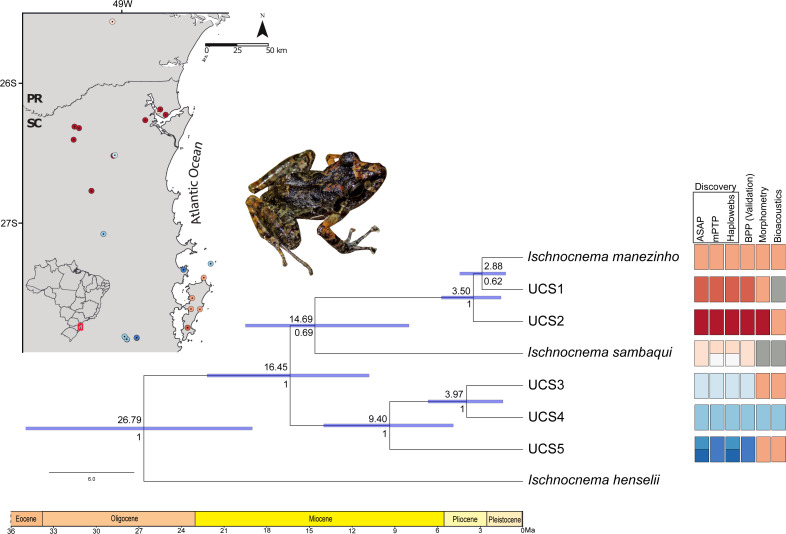
Time-calibrated species tree with each UCS distribution and summary results of distinct species delimitation methods of *Ischnocnema manezinho-Ischnocnema sambaqui* complex. Numbers below and above the blue bars indicate node probability and the median dating of each clade, respectively. Blue node bars indicate the 95% highest posterior density (HPD) of estimated times, represented in millions of years ago (Ma). Colors represent the distinct lineages delimited by each method, and grey squares represent the lack of samples in that UCS/method. We removed from this figure the non-*Ischnocnema* outgroups used in the species tree estimation, to facilitate the visualization. The complete result can be seen in Fig. S3. Photo credit: André Ambrozio-Assis.

*Ischnocnema sambaqui* is nested within *Ischnocnema manezinho sensu lato* in the species tree (Fig. 4), but with a different topology when compared with the tree estimated by BPP. In species tree, the clade composed by *Ischnocnema manezinho*, UCS1 and UCS2 is sister of *I. sambaqui* (*i.e.,* ((((*I. manezinho*, UCS1), UCS2), *I. sambaqui*), ((UCS3, UCS4), UCS5))), while in BPP tree, *I. sambaqui* is sister of the clade composed by UCS3, UCS4, and UCS5 (*i.e.*, (((*I. manezinho*, UCS1), UCS2), (((UCS3, UCS4), UCS5), *I. sambaqui*))). However, the phylogenetic placement of *I. sambaqui* is poorly supported in the species tree (PP = 69.5%, Fig. 4). The first cladogenetic event on ingroup occurred in the middle Miocene, approximately 16.45 million years ago (Ma), while most lineages diverged in Pliocene (Fig. 4, Fig. S3). The lineages *Ischnocnema manezinho* and UCS1 are sisters and diverged in the early Pliocene, around 2.88 Ma (Fig. 4).

## Discussion

Our molecular results support the hypothesis of six evolutionarily independent lineages under the name of *I. manezinho*, therefore we suggest this taxon only applies to the individuals of Santa Catarina Island. However, despite our genetic results showing high support to differentiate five distinct candidate species, our morphometric and bioacoustics data do not validate all of them.

These data support solely the differentiation of *I. manezinho* from UCS2 and UCS4, although with morphometric and acoustic parameters boundaries overlap. Thus, the five candidate species remain unconfirmed and need to be taxonomically investigated for confirmation or not as new species. Although we adopted the consensus for validating the lineages, it is notable that UCS5 presents a high intraspecific genetic diversity and high divergence in nuclear fragments. The composition of this candidate species may be a result of an underestimation of the mPTP method and a low sample size since we analyzed only six individuals. Thus, we suggest further investigation of this candidate species using genomic markers and a wide sampling of individuals and phenotypic characters.

In this sense, our results point to UCS2 and UCS4 as the priority putative species for a taxonomic description since it was the only two that differed from *I. manezinho* in morphometric traits and UCS4 was the only one that differed in bioacoustics traits. Model-based and integrative methods of species delimitation can provide meaningful results for planning *in situ* conservation and management of the species, helping in decision-making about taxonomic identity and geographical distribution (Delić et al., 2017; Magalhães et al., 2018). For conservation purposes, it is indispensable that the species receive a formal name, which is mandatorily linked to diagnostic characters (Delić et al., 2017). So that this can happen in practice, a molecular putative species must be validated by integrative data, like morphology, behavior, or ecology (Fujita et al., 2012; Sukumaran & Knowles, 2017). Amphibians communicate mainly by means other than visual signals, favoring the maintenance of morphological homogeneity between related lineages (Bickford et al., 2007). In this context, bioacoustics should be evaluated as an alternative source of diagnostic characters since it is almost universally applied for anurans, and differences in advertisement calls can be interpreted as indirect evidence of reproductive isolation (see Köhler et al., 2017 for a review).

It is noteworthy that UCS4 occurs on mainland and on a continental island, called Arvoredo Island. Despite this island be much further away from the mainland than Santa Catarina Island—∼13 km *versus* ∼0.5 km, respectively—mainland and island population of UCS4 showed only 0.59% genetic divergence, while UCS1 and *I. manezinho* are approximately 10 times more divergent. This result evidences a very recent isolation of the population of Arvoredo Island. Sea level changes during the Pleistocene climatic fluctuations may have resulted in connectivity between this island and the mainland (*e.g.*, Leite et al., 2016). These hypotheses should be better investigated using ecological niche and sea level modeling.

The deep divergence times estimated in our study corroborate the taxonomic scenario of five putative species. The most recent common ancestor (MRCA) of the ingroup clade predates the Miocene and suggests ancient isolation between the internal clade of the *I. manezinho –I. sambaqui* complex. This divergence time is comparatively more recent than that found for the MRCA between *I.* cf. *manezinho* (= UCS2) and *I. sambaqui* by Taucce et al. (2018). This discrepancy could be attributed to differences in genetic markers sampling and divergence time estimation strategy. Despite these differences, there is an overlap between the credibility intervals of MRCA obtained by us (∼8.7–19.4 Ma) and Taucce et al. (2018) (∼13–32 Ma), both indicating deep divergence between the lineages. Furthermore, most cladogenetic events are old and predate Pliocene and those results contradict the assumption that morphologically similar species are always resultant of recent events of speciation (Bickford et al., 2007; Fišer, Robinson & Malard, 2018; Struck et al., 2018), pointing out that other factors, such, morphological convergence, parallelism, or stasis may be the mechanisms involved in the diversification of this group (Fišer, Robinson & Malard, 2018; Struck et al., 2018). The results show a possible paraphyly between *I. manezinho sensu lato* and *I. sambaqui*, which would favor the hypothesis that the external morphology similarities in *I. manezinho* lineages result from morphological parallelism or convergence (Fišer, Robinson & Malard, 2018; Struck et al., 2018). This hypothesis is reinforced by the fact that *I. sambaqui* differs from *I. manezinho* morph by larger size, head wider than long, presence of a heel tubercle, and external vocal sac, both absent in *I. manezinho* (Garcia, 1996; Castanho & Haddad, 2000). This could be associated with distinct habitat uses since *I. manezinho sensu lato* is rupicolous while *I. sambaqui* is arboricolous (CBO, PCAG, pers. obs, 2016). However, we cannot discard the hypothesis that divergent selection resulted in morphological and behavioral distinctness in *I. sambaqui*, but this should be evaluated in a broader phylogenetic context (Struck et al., 2018). Nevertheless, the phylogenetic placement of *I. sambaqui* is not strongly supported, and the inclusion of additional independent markers in the phylogenetic reconstruction may solve this issue, increasing the species tree estimation accuracy (Knowles & Kubatko, 2011).

All lineages showed high pairwise genetic distances in mtDNA (5.58–28.77%), similar to or even higher than compared to those found between other *Ischnocnema* groups, like *I. venancioi* (Taucce et al., 2018) and *I. guentheri* series (Gehara et al., 2013; Taucce, Canedo & Haddad, 2018). However, these results should be compared with caution, because the uncorrected p-distances in the literature were calculated from rDNA 16S, which tends to have much less variation than cyt-*b* (Caccone et al., 1997). The strong genetic structure and the restricted geographic distribution, as observed in the *Ischnocnema manezinho –I. sambaqui* species complex, were reported in several species of anurans from distinct regions (*e.g.*, Fouquet et al., 2007; Funk, Caminer & Ron, 2012; Vacher et al., 2020), including species of the same genus spread over the Atlantic Forest (Gehara et al., 2013; Gehara et al., 2017; Taucce, Canedo & Haddad, 2018; Taucce et al., 2018). This may indicate that many species known as widely distributed may be a mosaic of undescribed diversity, harming species-focused conservation (Vacher et al., 2020).

Our results are not free of caveats. Although we have evaluated phenotypic traits, they are not congruent with the different lineages found in the genetic data. Thus, we reinforce that new independent data is needed for a complete taxonomic review of the species complex. Morphometry does not show a taxonomic signal for *I. manezinho sensu lato*. However, external qualitative characters, osteological, muscular, and/or visceral anatomy can be important sources of diagnostic characteristics for anurans, including other brachycephalids (Guimarães et al., 2017). It is noteworthy that, besides morphology, vocalizations were already used for diagnosing other *Ischnocnema* species (Taucce, Canedo & Haddad, 2018; Taucce et al., 2018), in addition to other species of anurans (Hepp et al., 2015). We evaluated only four traits in the pairwise test since some parameters showed a collinearity problem. We also evaluated a single type of call (*i.e.,* advertisement call) and only for four candidate species with few individuals, hence it may be important to assess calls emitted in other contexts than female attraction.

Our results reveal several candidate species with limited geographic distributions, making each of them more prone to extinction by stochastic events or inbreeding (Bickford et al., 2007). Until they receive a formal name, they should be treated as independent lineages for conservation. Despite the importance of lineage conservation for maintaining the evolutionary potential of species (Fraser & Bernatchez, 2001), these intraspecific units are not considered in Brazilian conservation policies (Magalhães et al., 2017). It is worrisome because if the *I. manezinho*’s lineages are not correctly managed, millions of years of accumulated evolutionary history can be lost through local extinctions, especially in fragmented areas.

Following the evolutionary concept of species (Simpson, 1951), our approach allows us to observe evolutionary independence between the proposed species through multiple criteria, like reciprocal monophyly, gene flow barriers, and allele exclusivity (de Queiroz, 2007), helping in Red List assessments. The last Brazilian Red List (MMA, 2022) used the unpublished results and preliminary data from this study to consider only Santa Catarina Island populations in the evaluation of *Ischnocnema manezinho*. However, the management of *Ischnocnema manezinho* and UCS1, for example, should be made considering the potential effects of crosses between them, which depending on their differentiation level, could either result in consequences as distinct as heterosis (adaptive introgression) or outbreeding depression (Frankham et al., 2012). Because these lineages present high genetic divergence between them and are allopatric, the deleterious effect of outcrossing (outbreeding depression) is a likely management outcome. Additionally, our results suggest that non-forest areas are not suitable for *I. manezinho*, and these areas can be acting as a barrier to gene flow between the two delimited lineages (*i.e.*, *I. manezinho* and UCS1). When the two lineages are considered as separate taxa, following our results, we saw that the UCS1 is distributed in a very restricted area.

*Ischnocnema manezinho* and UCS1 suffer from common threats to coastal environments in Brazil, such as the continuous urbanization process (ICMBio, 2014). Although two of the four occurrence records of *I. manezinho* and the only record of UCS1 coincide with a municipal protected area, they do not fit into the National System of Protected Areas (SNUC; MMA, 2000), allowing several human activities and making the long-term protection the natural habitats unfeasible (IUCN, 2012; IUCN, 2017). In this scenario, UCS1 should be preserved as a Critically Endangered lineage, revealing a greater risk of local extinction when compared with the *I. manezinho*. However, if the UCS1 is not confirmed as a candidate species, preserving the two lineages as isolated units will lead to inbreeding, which would increase the risk of extinction of each of them (Frankham et al., 2012). Therefore, crossing experiments between individuals of the two lineages should be made to assess the likely outcome (outbreeding depression or heterosis) and evaluate the feasibility of translocations between each lineage area (Frankham et al., 2012). Additionally, the monitoring of *I. manezinho*, with particular attention to the UCS1, must be made to ensure the probability of the long-term persistence of the species. So, we highlight the importance of integrative delimitations in fauna assessments, for an accurate assessment and effective species conservation actions. For example, our previous results supported a law for naming *Ischnocnema manezinho* as a symbol species for the municipality of Florianópolis. The municipal category will allow legal, public, and collective actions in favor of the conservation of this species.

## Conclusion

Despite the lack of morphometric and call traits for diagnosing most of the *I. manezinho* lineages, the deep genetic differentiation between them, the paraphyly of *I. manezinho sensu lato* concernig to *I. sambaqui*, and the morphometric and bioacoustics distinctness between UCS4 and others UCS reinforce the hypothesis of multiple species. Our model-based DNA approach strongly supports that *I. manezinho* is endemic to Santa Catarina Island, even in the absence of diagnoses data for the five candidate species, distributed in the continental portion of Santa Catarina State. However, this work does not solve the taxonomic problem of *I. manezinho* species complex. A detailed taxonomic investigation is a research priority, focusing on other character systems than those used here. Finally, the results show the importance of model-based taxonomy to define geographic limits in species complexes, improving its taxonomy, threat categorization, and management.

##  Supplemental Information

10.7717/peerj.15393/supp-1 Supplemental Information 1 Results of ASAP (Assemble Species by Automatic Partitioning) method (A) The dendrogram and the best delimitation scheme. (B) The histogram of distances, showing the barcode gap, and (C) the rank of distances. The red line represents the distance threshold that indicates the best partition for the hypothesis of the species delimitation.

10.7717/peerj.15393/supp-2 Supplemental Information 2 Fields for Recombination for each fragment marker. (A) Fibrinogen A alpha-polypeptide, intron 1 (*α*-fib). (B) Beta-fibrinogen, intron 7 (*β*-fib). (C) Chemokine Receptor 4 (*cxc*)

10.7717/peerj.15393/supp-3 Supplemental Information 3 Time-calibrated species tree Numbers below and above the blue bars indicate node probability and the median dating of each clade, respectively. Blue node bars indicate the 95% highest posterior density (HPD) of estimated times, represented in millions of years ago (Ma).

10.7717/peerj.15393/supp-4 Supplemental Information 4 Supplementary Tables

## Additional Information and Declarations

### Field Study Permissions

The following information was supplied relating to field study approvals (i.e., approving body and any reference numbers): The Instituto Chico Mendes de Conservação da Biodiversidade (ICMBio) and Instituto do Meio Ambiente de Santa Catarina (IMA) approved thist research (47781, 45770, 12/2015).

### DNA Deposition

The following information was supplied regarding the deposition of DNA sequences: The gene sequences are available at GenBank: OP185393 to OP185447; OP185448 to OP185485; OP270087 to OP270119, and OP270120 to OP270157.

### Data Availability

The following information was supplied regarding data availability: The raw data, specimens, localities, geographic coordinates, voucher ID and herpetological collections are available in the Supplemental Files.
