# Supplementary figures and images for: Integrative species delimitation helps to find the hidden diversity of the leaf-litter frog Ischnocnema manezinho (Garcia, 1996) (Anura, Brachycephalidae), endemic to the southern Atlantic Forest
Source: PeerJ. 2023 May 25;11:e15393. doi: 10.7717/peerj.15393 (PMC10225124; doi:10.7717/peerj.15393)

A

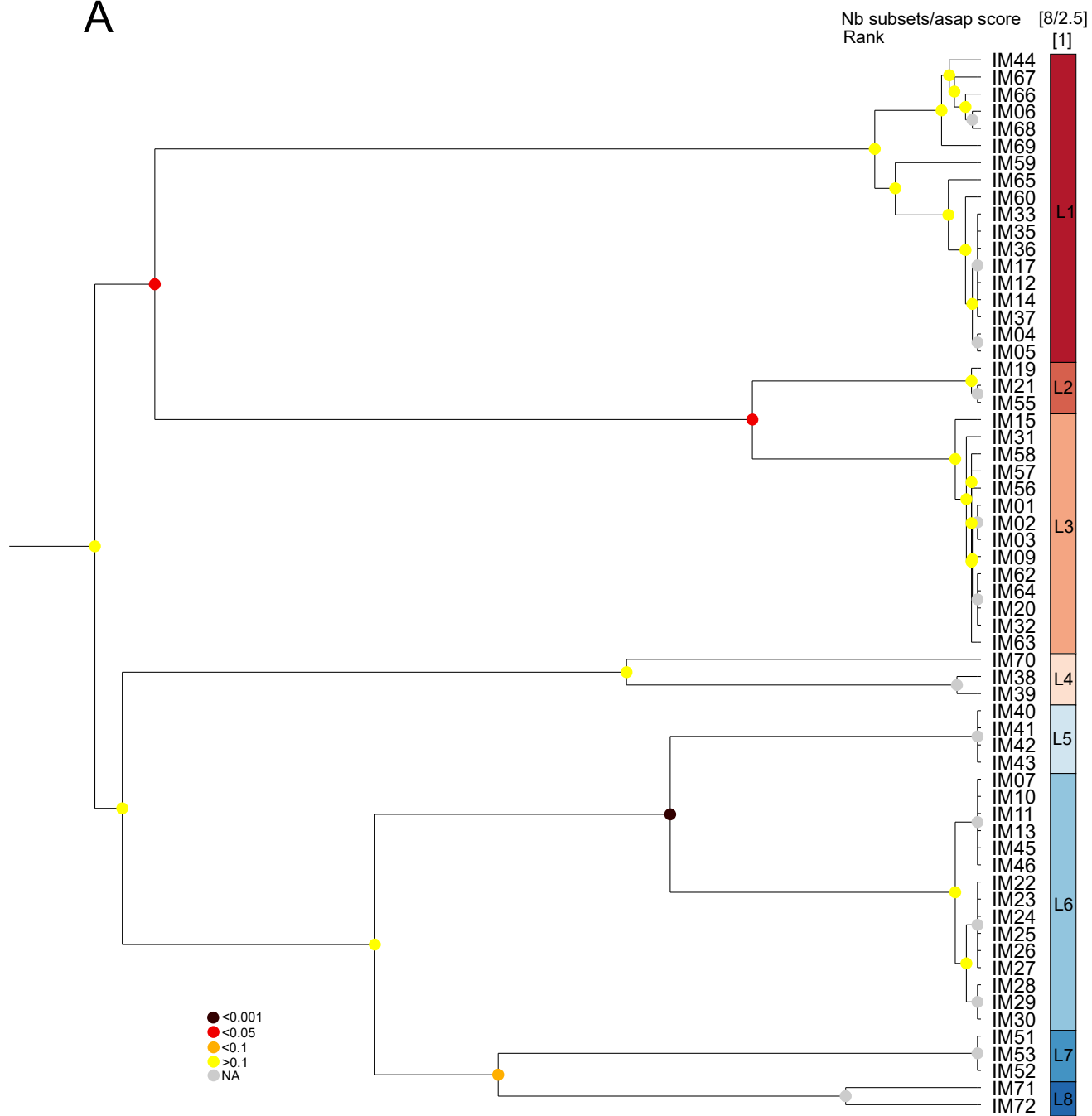

B

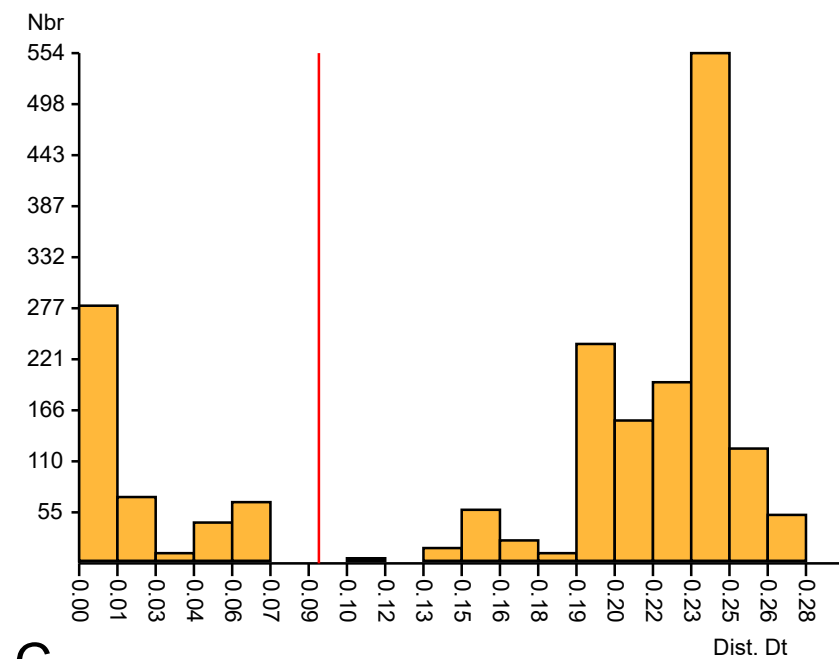

C

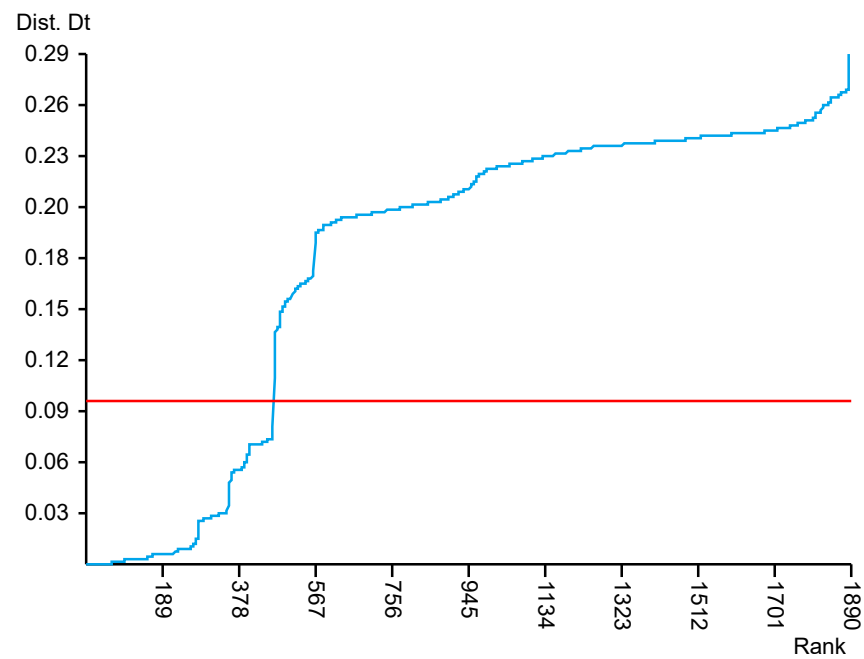

Supplement: Supplemental Information 1 — (A) The dendrogram and the best delimitation scheme. (B) The histogram of distances, showing the barcode gap, and (C) the rank of distances. The red line represents the distance threshold that indicates the best partition for the hypothesis of the species delimitation. [file peerj-11-15393-s001.pdf]

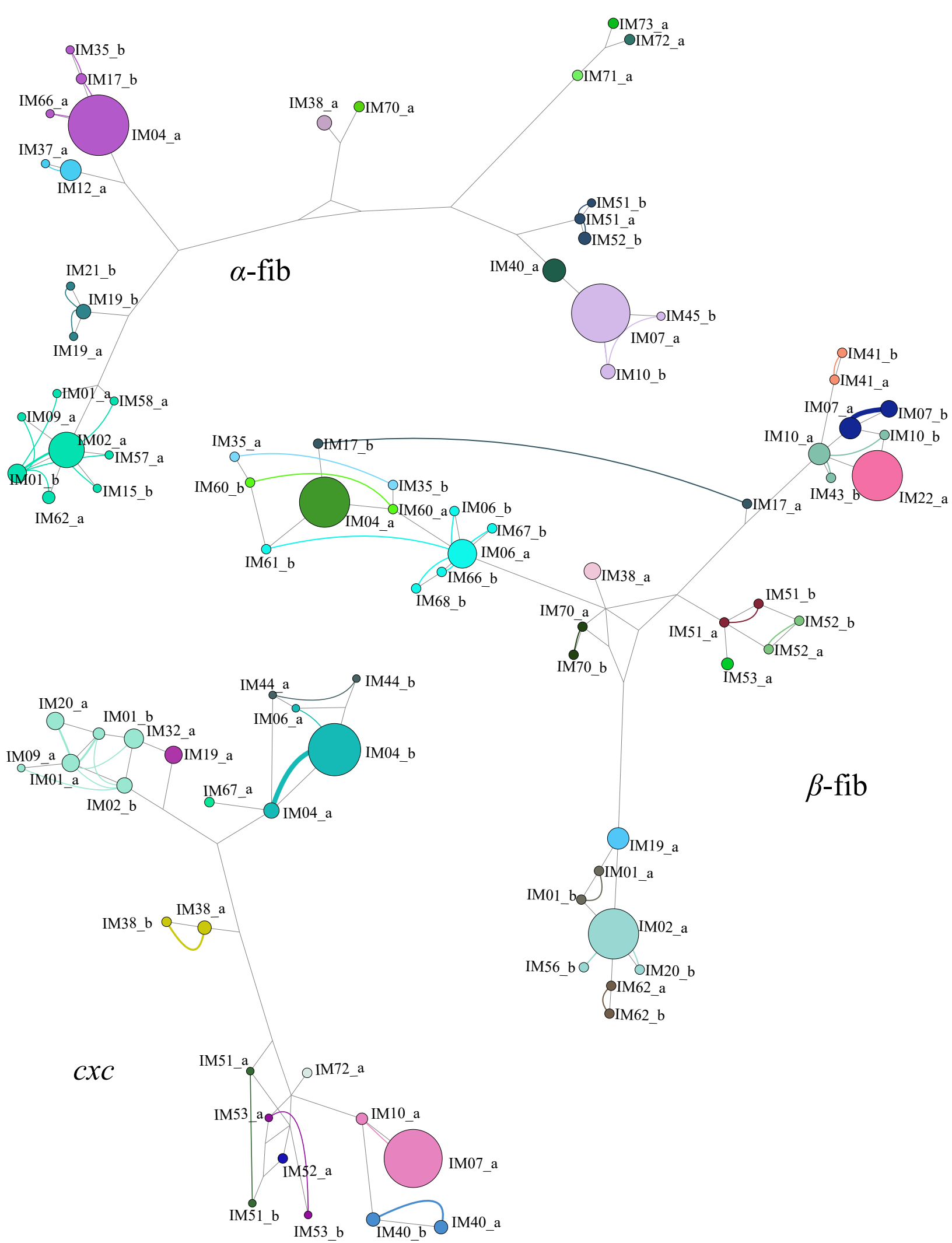

Supplement: Supplemental Information 2 [file peerj-11-15393-s002.pdf]

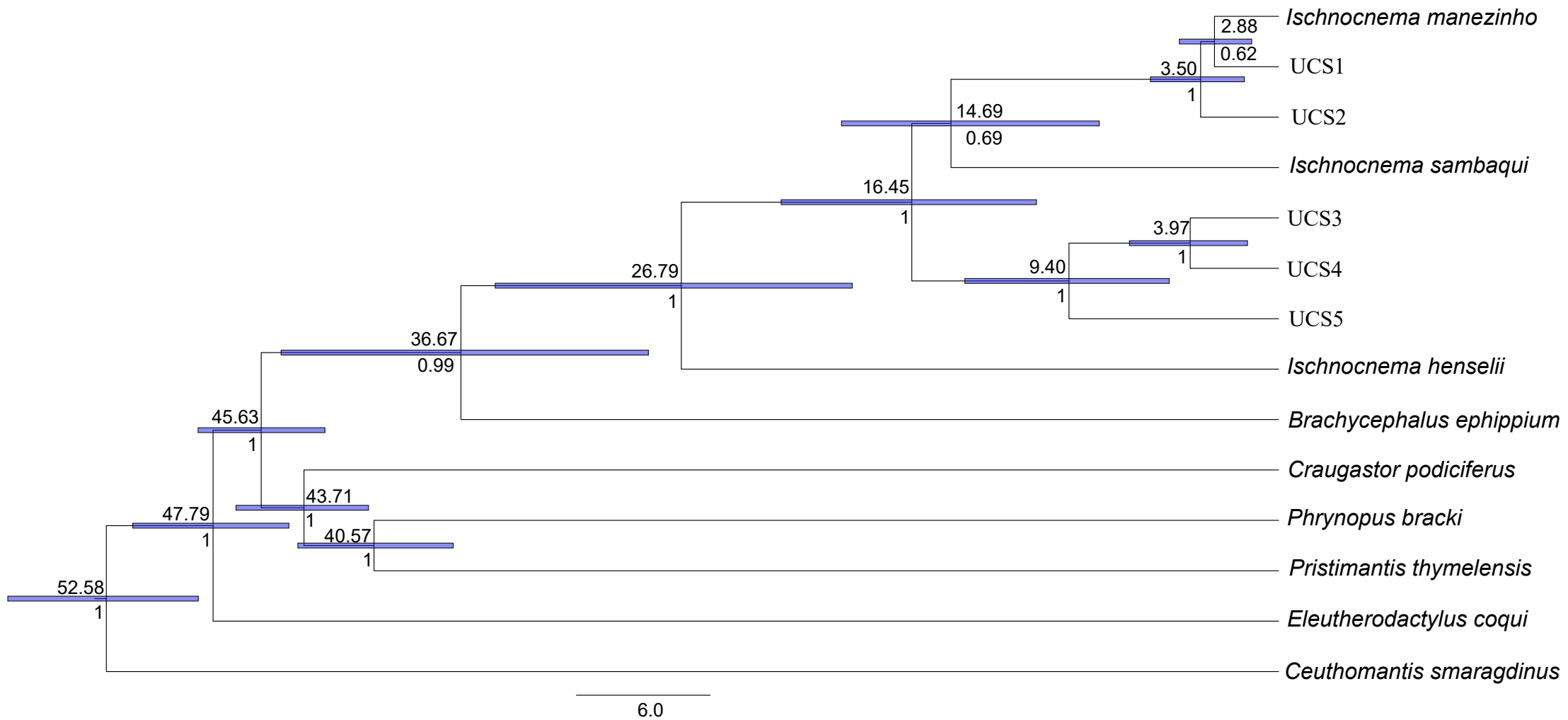

Supplement: Supplemental Information 3 — Numbers below and above the blue bars indicate node probability and the median dating of each clade, respectively. Blue node bars indicate the 95% highest posterior density (HPD) of estimated times, represented in millions of years ago (Ma). [file peerj-11-15393-s003.pdf]
